# Input Convergence, Synaptic Plasticity and Functional Coupling Across Hippocampal-Prefrontal-Thalamic Circuits

**DOI:** 10.3389/fncir.2018.00040

**Published:** 2018-05-23

**Authors:** Lezio S. Bueno-Junior, Joao P. Leite

**Affiliations:** Department of Neuroscience and Behavioral Sciences, Ribeirão Preto Medical School, University of São Paulo, Ribeirão Preto, Brazil

**Keywords:** unit activity, field potentials, electrical brain stimulation, optogenetics, fear conditioning, decision making, spatial navigation, anxiety

## Abstract

Executive functions and working memory are long known to involve the prefrontal cortex (PFC), and two PFC-projecting areas: midline/paramidline thalamus (MLT) and cornus ammonis 1 (CA1)/subiculum of the hippocampal formation (HF). An increasing number of rodent electrophysiology studies are examining these substrates together, thus providing circuit-level perspectives on input convergence, synaptic plasticity and functional coupling, as well as insights into cognition mechanisms and brain disorders. Our review article puts this literature into a method-oriented narrative. As revisited throughout the text, limbic thalamic and hippocampal afferents to the PFC gate one another’s inputs, which in turn are modulated by PFC interneurons and ascending monoaminergic projections. In addition, long-term synaptic plasticity, paired-pulse facilitation (PPF), and event-related potentials (ERP) dynamically vary across PFC-related circuits during learning paradigms and drug effects. Finally, thalamic-prefrontal loops, which have been shown to amplify both cognitive processes and limbic seizures, are also being implicated as relays in the prefrontal-hippocampal feedback, contributing to spatial navigation and decision making. Based on these issues, we conclude the review with a critical synthesis and some research directions.

## Introduction

The ability to control behavioral actions upon environmental demands is critical for survival and social acceptance. Failing to do so can become maladaptive, or, in the case of humans, evolve into psychiatric symptoms. Such a cognitive control, i.e., executive functions, could not simply rely on discrete brain sites, but rather on an extensive multimodal network. This network crucially involves the prefrontal cortex (PFC) and two PFC-projecting areas: the midline/paramidline thalamus (MLT; Vertes et al., 2015) and the hippocampal formation (HF; Verwer et al., 1997). Revisiting this circuit is the aim of our review article, with an emphasis on rodent electrophysiology studies that examined the three substrates together. As implied by the intersection of thalamic and hippocampal terminal fields in the medial PFC of rodents (mPFC; Gigg et al., 1994; Floresco and Grace, 2003; Hugues and Garcia, 2007; Bolkan et al., 2017), as well as the prefrontal-thalamic-hippocampal coupling in goal-directed behaviors (Ito et al., 2015; Hallock et al., 2016), these limbic sites cooperate for an adaptive cognitive control.

First, an anatomical overview is presented based on tract tracing and cell/fiber labeling data from rodents. Second, a review is provided on rodent electrophysiology studies directly evidencing the hippocampal, prefrontal and thalamic cooperation. Lastly, research directions are outlined along with a critical synthesis. Influences to and from related limbic structures, such as the amygdala and ventral tegmental area (VTA), are mentioned throughout the text. Partially overlapping reviews are available on this topic. They are either focused on mPFC sub-regions and their interconnections through the ventral midline thalamus (Vertes, 2006; Cassel et al., 2013; Cassel and Pereira de Vasconcelos, 2015; Griffin, 2015), the functional heterogeneity of the thalamus as implied by rodent lesion and human diencephalic pathology studies (Mair et al., 2015; Wolff et al., 2015), or broad mechanisms of sleep-wake cycle and memory persistence (Pereira de Vasconcelos and Cassel, 2015). However, none of them delimited electrophysiological studies that manipulated/recorded all three brain sites in the same experiment, or set of experiments. Offering such a scope while potentially assisting experiment designing is the aim of our review article.

### Search Methods

Using PubMed, we combined “prefrontal” with thalamus- and hippocampus-related terms (e.g., “midline,” “mediodorsal,” “reuniens,” “cornus ammonis 1 (CA1),” “subiculum”), and then looked for rodent electrophysiology studies. Searches were narrowed to Title/Abstract, with the “Other Animals” filter on. As a result, we identified 31 methodologically pertinent articles, ~74% of which published since 2011 (Table 1). These articles are discussed in the main section of this text (“Rodent Electrophysiology Studies”), along with supporting citations.

**Table 1:** Methodological overview of the delimited literature (31 articles).

Reference	Strategy	Preparation	Recording	Behavior	Category
Gigg et al. (1994)	affer stimul, drug iontophor	anesth	mPFC firing		convergence
O’Donnell and Grace (1995)	curr injection, affer stimul^1^	anesth	NAc EPSP		convergence
Finch (1996)	affer stimul, retrogr tracing	anesth	NAc/CPu EPSP, firing		convergence
Giacchino and Henriksen (1998)	affer stimul, drug iontophor	anesth	mPFC firing		convergence
Lewis and O’Donnell (2000)	affer stimul, ip/ic drugs	anesth	mPFC EPSP, firing		convergence
O’Donnell et al. (2002)	affer stimul, hippoc lesion	anesth	mPFC EPSP, firing		convergence
Floresco and Grace (2003)	affer stimul, iv drugs	anesth	mPFC firing		convergence
Hugues and Garcia (2007)	affer stimul, LFS	chronic	mPFC fPSP	fear extinction	plasticity
Eleore et al. (2011)	affer stimul, HFS, PPR	chronic	mPFC, CA1 fPSP^1^	eyeblink condit^1^	plasticity
Kiss et al. (2011a)	affer stimul, PPR, ic/iv drugs	anesth	mPFC fPSP, LFP		plasticity
Kiss et al. (2011b)	affer stimul, PPR, ic/iv drugs	anesth	mPFC fPSP, LFP, firing		plasticity
Sloan et al. (2011a)	affer stimul, kindl, ic TTX	anesth	mPFC, EC fPSP, AD		plasticity
Sloan et al. (2011b)	affer stimul, kindl, ic muscim	anesth	MD, mPFC fPSP, AD		plasticity
Little and Carter (2012)	affer optogen stimul^1^	*in vitro*	mPFC EPSC, Ca^2+^ signals^1^		plasticity
Calhoon and O’Donnell (2013)	affer stimul, intracell picrot	anesth	NAc/CPu EPSP		plasticity
Parnaudeau et al. (2013)	chemogen inhib	chronic	MD, mPFC, hippoc	DNMS T-maze^1^	coupling
Grupe et al. (2014)	sensory stimul, po drugs	chronic	multi-site ERP	auditory discrimin	plasticity
Ito et al. (2015)	traj-depend act, optogen control^1^	chronic	CA3–1, Re, mPFC firing^1^	modified T-mazes^1^	coupling
López-Ramos et al. (2015)	affer stimul, HFS, PPR	chronic	mPFC fPSP	food/shock decision	plasticity
Hallock et al. (2016)	perf-depend act, ic muscim^1^	chronic	CA1 LFP, mPFC firing	modified T-mazes	coupling
Hartung et al. (2016)	ic lidocaine, retrogr tracing^1^	anesth	multi-site LFP, firing		coupling
Kjaerby et al. (2016)	affer optogen control, PPR^1^	*in vitro*, chronic	mPFC EPSC	EPM^1^	convergence
Liu et al. (2016)	affer optogen control, PPR^1^	*in vitro*, chronic	NAc EPSC	sucr self-admin^1^	convergence
Padilla-Coreano et al. (2016)	affer optogen inhib	chronic	vHipp, BLA, mPFC	EPM, open field^1^	coupling
Zimmerman and Grace (2016)	multi-site ic drugs	anesth^2^	VTA firing	open field^2^	coupling
Bolkan et al. (2017)	affer optogen inhib	chronic	MD LFP, mPFC firing	DNMS T-maze	coupling
Hernández-González et al. (2017)	brain-machine interface	chronic	multi-site LFP	operant condit	coupling
Jett et al. (2017)	affer stimul, chronic stress^1^	anesth^2^	mPFC I/O curve, c-Fos^1^	attent set-shifting^2^	plasticity
Roy et al. (2017)	pontine stimul, ic lidocaine^1^	anesth	mPFC, hippoc, Re LFP		coupling
Bueno-Junior et al. (2018)	HFS, PPR, optogen control^1^	chronic, anesth	mPFC, PV/MD fPSP, firing		coupling
Kafetzopoulos et al. (2018)	Re lesion, stress, dendr morphol^1^	anesth^2^	mPFC, CA1 LFP	forced swim, sucr pref	coupling

*^1^Indicates that other methods have been omitted in the Table. ^2^Indicates separate experiments for anesthetized recordings and behavioral testing. Abbreviations: act, activity; AD, after-discharges; affer, afferent; anesth, anesthetized; attent, attentional; BLA, basolateral amygdala; CA1, cornus ammonis 1; condit, conditioning; CPu, caudate-putamen; curr, current; dendr, dendritic; dep, deprivation; discrimin, discrimination; DNMS, delayed nonmatch-to-sample; EC, entorhinal cortex; EPSC, excitatory postsynaptic currents; EPSP, excitatory postsynaptic potentials; ERP, event-related potentials; chemogen, chemogenetic; fPSP, field postsynaptic potentials; HFS, high-frequency stimulation; hippoc, hippocampus; ic, intracerebral; inhib, inhibition; intracell, intracellular; I/O, input-output; iontophor, iontophoresis; ip, intraperitoneal; iv, intravenous; kindl, kindling; LFP, local field potentials; LFS, low-frequency stimulation; MD, mediodorsal thalamus; morphol, morphology; mPFC, medial prefrontal cortex; muscim, muscimol; NAc, nucleus accumbens; optogen, optogenetic; perf-depend, performance-dependent; picrot, picrotoxin; po, peroral; PPR, paired-pulse ratio; PV, paraventricular thalamus; Re, reuniens nucleus; retrogr, retrograde; self-admin, self-administration, stimul, stimulation; sucr, sucrose; traj-depend, trajectory-dependent; TTX, tetrodotoxin; vHipp, ventral hippocampus; VTA, ventral tegmental area. Table organized as in Ruggiero et al. (2017)*.

## Anatomical Overview

The initial criterion for defining the PFC, i.e., projecting area of the mediodorsal thalamus (MD; Rose and Woolsey, 1948; Leonard, 1969; Guldin et al., 1981; Divac et al., 1993), has been updated by functional, developmental and cytoarchitectonic observations (Groenewegen et al., 1997; Uylings et al., 2003). In addition, increasing importance is given to cortico-cortical connectivity when delineating the PFC, its subdivisions, and the homology of PFC subdivisions between rodents and primates (Fuster, 2015). This includes the partial homology between the rodent mPFC and the primate dorsolateral PFC, which is a well accepted framework for understanding executive functions (Vertes, 2006). In this review article, we give focus to the rodent mPFC, which is known to interact with multiple thalamic areas. These areas are collectively referred to as MLT, and comprise the paraventricular (PV), paratenial (PT), central medial (CM), interanteromedial (IAM), intermediodorsal (IMD), rhomboid (Rh), and reuniens (Re) nuclei, as well as the medial portion of the MD (Vertes et al., 2015). Despite some overlap among terminal fields, each of these nuclei gives rise to its own topography of predominantly ipsilateral projections, with specific distribution patterns across cortical areas and layers (Berendse and Groenewegen, 1991; Hoover and Vertes, 2007).

The most studied sources of thalamic outputs to the mPFC reside in the dorsal (MD, PV, PT) and ventral (Rh, Re) regions of the MLT. In particular, the basic topography of the MD-mPFC connectivity is organized across the MD medial-lateral extent and the mPFC dorsal-ventral extent, and includes different innervation patterns from limbic subcortical sites, like the amygdala and ventral pallidum (Krettek and Price, 1977; Groenewegen, 1988; Ray and Price, 1992; Wang and Shyu, 2004; Alcaraz et al., 2016; Kuramoto et al., 2017). Similarly to the MD, the PV and PT nuclei have also been shown to target the mPFC, as well as several limbic areas, including the subiculum (Sub), but not the hippocampus proper (Vertes and Hoover, 2008; Mátyás et al., 2014). In this sense, the ventral midline thalamus has attracted particular interest, because the Rh and Re nuclei have been found to directly innervate the hippocampus proper, namely CA1, in addition to the Sub, mPFC and widespread limbic sites (Dolleman-Van Der Weel and Witter, 1996; Vertes et al., 2006). Importantly, retrograde tracing studies have demonstrated a proportion (<10%) of Re cells sending axon collaterals to both the mPFC and hippocampus, implicating the Re nucleus in activity synchronization (Hoover and Vertes, 2012; Varela et al., 2014).

Similarly to other thalamocortical systems, the mPFC is known to reciprocate its thalamic afferents (Cornwall and Phillipson, 1988; Ray et al., 1992; Vertes, 2002; Li and Kirouac, 2012). This connectivity includes MD axons terminating on MD-projecting pyramidal cells of the mPFC (Kuroda et al., 1993, 1998). The same rationale applies to back projections from CA1 and Sub to the Re nucleus, indicating that the ventral midline thalamus relays a two-way connection between the HF and the mPFC (McKenna and Vertes, 2004; Vertes et al., 2007; Prasad and Chudasama, 2013). The bidirectional transthalamic route between the HF and the mPFC contrasts with the unidirectional hippocampal-prefrontal projections. In fact, the temporal CA1-mPFC pathway had been the first demonstrated link between the hippocampus proper and a distant neocortical area (Swanson, 1981; Ferino et al., 1987; Jay et al., 1989). The CA1-bordering portion of the Sub, which itself receives inputs from CA1 (Tamamaki et al., 1987; Amaral et al., 1991), also contribute fibers to CA1-innervated areas of the mPFC (Jay and Witter, 1991), explaining why the CA1/Sub area is commonly approached as an integrated source of hippocampal outputs (Jay et al., 1996; Laroche et al., 2000; Thierry et al., 2000; Nakamura et al., 2010; Godsil et al., 2013). Finally, it is also worth mentioning that the mPFC is among the projection areas of the entorhinal cortex (EC; Swanson and Köhler, 1986; Condé et al., 1995), which similarly to the ventral midline thalamus is known to mediate cortico-hippocampal flows of information (Insausti et al., 1997; Canto et al., 2008).

**Figure 1:**
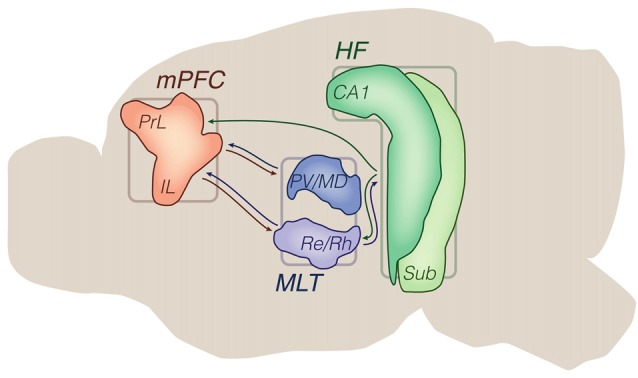
Principal brain areas and axonal projections of the delimited literature. Mid sagittal view of the rodent brain. Other brain sites are illustrated in subsequent figures (e.g., ventral tegmental area (VTA), basolateral amygdala (BLA), striatum). Arrows from the hippocampal formation (HF) are generically positioned, i.e., no distinction is made between dorsal, intermediate and ventral domains. Drawings are based on the Brain Explorer 3D atlas (Allen Institute). Abbreviations: CA1, cornus ammonis 1; HF, hippocampal formation; IL, infralimbic area; MLT, midline/paramidline thalamus; mPFC, medial prefrontal cortex; PrL, prelimbic area; paraventricular (PV)/mediodorsal thalamus (MD), PV/mediodorsal nuclei; Re/Rh, reuniens/rhomboid nuclei; Sub, subiculum.

In general, the rodent HF, limbic thalamus and mPFC interact with each other through topographically complex connectivity patterns, which can nevertheless be simplified into a tripartite system (Figure 1). Based on this map, we now review electrophysiological works that manipulated/recorded the three substrates in a cohesive manner.

## Rodent Electrophysiology Studies

### Input Convergence: Relationships With the Mesocorticolimbic System

Gigg et al. (1994) were the first to directly evidence the functional convergence of limbic thalamic and hippocampal inputs to the mPFC. In chloral hydrate-anesthetized rats, the authors extracellularly recorded single-unit activity from the mPFC while delivering electrical paired pulses into ipsilateral thalamic and hippocampal areas, including the medial MD and temporal CA1/Sub. Among other results, a proportion of CA1/Sub-recruited neurons in the mPFC (six of 16) also responded to MD thalamic stimulation, and both responses could be blocked by iontophoretic administration of CNQX (AMPA receptor antagonist). Furthermore, application of bicuculline (GABA_A_ receptor antagonist) revealed excitatory responses in a subset of neurons that were otherwise unresponsive to hippocampal pulses. Thus, the findings of Gigg et al. (1994) indicated that thalamic and hippocampal afferent convergence in the mPFC is partially at the single-cell level, is mediated by glutamate transmission, and is modulated by local GABAergic transmission, which would be later reinforced by observations of thalamic (Kuroda et al., 2004; Rotaru et al., 2005) and hippocampal (Tierney et al., 2004; Takita et al., 2007) terminals onto mPFC interneurons. A similar work in halothane-anesthetized rats (Giacchino and Henriksen, 1998) reproduced the mPFC firing responses to MD thalamic and CA1/Sub electrical stimuli, this time under systemic morphine. The authors reported a predominantly inhibitory (and naloxone-reversible) action of morphine on evoked mPFC firing, especially when the MD was stimulated. While specifically implicating the opioid modulation on afferent-driven mPFC activity, the results of Giacchino and Henriksen (1998) suggest that this and other neuromodulatory systems may exert differential effects throughout mPFC inputs. Indeed, the same study examined another mPFC-projecting structure, the basolateral amygdala (BLA), whose inputs to the mPFC were also sensitive to morphine, but only to a lesser degree.

In subsequent studies, other sources of mPFC inputs have been examined in addition to the HF and limbic thalamus: VTA, BLA, or the contralateral mPFC (Lewis and O’Donnell, 2000; O’Donnell et al., 2002; Floresco and Grace, 2003; Little and Carter, 2012; Kjaerby et al., 2016). Using chloral hydrate-anesthetized rats, Lewis and O’Donnell (2000) described how electrical pulses into the VTA, ventral Sub, MD thalamus, or fimbria-fornix (a bundle of hippocampal fibers) affect the membrane potential of mPFC pyramidal cells. Although hippocampal and thalamic inputs could disrupt membrane potentials, only VTA stimuli, especially burst-like ones, could drive lasting depolarization states depending on the pre-stimulus membrane potential. As speculated by Lewis and O’Donnell (2000), these action potential-enabling “up” states induced by VTA stimulation would facilitate NMDA receptor-dependent synaptic plasticity, while providing time frames of increased sensitivity to inputs. Subsequently, O’Donnell et al. (2002) showed that neonatal lesions in the ventral hippocampus (vHipp) alter the mPFC responsivity to VTA, but not MD, stimulation during adulthood. These interactions were then examined in greater detail in urethane-anesthetized rats by Floresco and Grace (2003). The authors found that mPFC cells show different firing responses to MD and fimbria-fornix pulses depending on how they are timed (either MD then fimbria-fornix, or fimbria-fornix then MD), and their inter-stimulus interval (from 10 ms to 500 ms). In addition, they found that mPFC responses to fimbria-fornix pulses are modulated in complex manners by conditioning burst-like stimuli in the MD or VTA. First, these results confirm the previously known anatomical convergence of MD and VTA afferents onto mPFC pyramidal cells (Kuroda et al., 1996). Second, they reinforce, along with Giacchino and Henriksen (1998), the interplay of glutamatergic mPFC-centered pathways with other neurotransmitter systems. Most importantly, this set of findings reveals temporally precise relationships between dopaminergic and glutamatergic inputs to the mPFC, implying complex gating actions of mesolimbic structures on cognitive operations.

A decade later, thalamic and hippocampal input convergence in the mPFC has gained a deeper understanding thanks to optogenetics and synaptic physiology tools. Using whole-cell recordings from mouse mPFC *in vitro*, Little and Carter (2012) stimulated channelrhodopsin-expressing presynaptic terminals from either MD, vHipp, BLA, or the contralateral mPFC (i.e., callosal projections). All afferents evoked excitatory postsynaptic currents (EPSCs) in layer 2 pyramidal neurons, which could be blocked by NMDA and AMPA receptor antagonists. At the subcellular level, the authors observed calcium signals upon afferent stimulation, with each input activating a distinct population of dendritic spines. Because of the BLA stimulation (Little and Carter, 2012), these findings suggest a link between emotional processing and mPFC input convergence. A comparable link was explored by Kjaerby et al. (2016) through optogenetic stimulation of the same pathways—except BLA-mPFC—and presynaptic serotonergic modulation *in vitro*. According to the authors, layer 5 prefrontal EPSCs driven by callosal and vHipp, but not MD, projections were suppressed by serotonin acting on presynaptic 5-HT1B receptors. In a complementary *in vivo* experiment, Kjaerby et al. (2016) found that intra-mPFC 5-HT1B agonism diminishes both innate anxiety—as measured via the elevated plus maze (EPM)–and 4–30 Hz local field potential (LFP) oscillations, which include theta and beta bands. Therefore, the Kjaerby et al. (2016) findings add to those of Lewis and O’Donnell (2000), O’Donnell et al. (2002) and Floresco and Grace (2003) in supporting monoaminergic roles on the mPFC afferent drive.

In a final set of studies, the limbic striatum, rather than the mPFC, has been assessed as the converging node (O’Donnell and Grace, 1995; Finch, 1996; Calhoon and O’Donnell, 2013; Liu et al., 2016). In a work aimed at describing the nucleus accumbens (NAc) membrane properties under chloral hydrate anesthesia, O’Donnell and Grace (1995) described NAc single-cell responses to PV thalamic and, most prominently, mPFC, fimbria-fornix, and amygdala stimuli. A year later, Finch (1996) further explored the input convergence to the ventral striatum, similarly to what had been done by the same research group with the mPFC (Gigg et al., 1994). According to Finch (1996), stimulation of different sites recruited different proportions of medium spiny neurons at the NAc core/caudate-putamen (CPu) vicinity. In descending order of evoking efficacy, the sites were the mPFC, CM thalamus, EC, BLA and ventral CA1/Sub. Pairs of these sites were also shown to evoke convergent responses at the single-cell level; for example, 29% of medium spiny neurons reacted to both mPFC and CM pulses, whereas 9% responded to both EC and ventral CA1/Sub.

Almost two decades later, the timing between ventral striatum inputs was explored by Calhoon and O’Donnell (2013) in chloral hydrate-anesthetized rats. According to the findings, conditioning stimulation of the mPFC with 50-Hz trains suppressed fimbria-fornix-evoked field postsynaptic potentials (fPSPs) in the ventral striatum. However, this suppression was only effective with a short latency between mPFC and fimbria-fornix stimuli (50 ms), while at long latency (500 ms) fimbria-fornix-evoked fPSPs were unaffected. This result was basically replicated when a thalamic nucleus (dorsolateral) was stimulated instead of the fimbria-fornix. Thus, the mPFC throughput seems to regulate the ventral striatal sensitivity to other afferents in a timing-dependent manner, which could represent the prefrontal control over basal ganglia and related behaviors (Calhoon and O’Donnell, 2013). This descending influence from the mPFC has more recently been associated with sleep in mice (Liu et al., 2016). Through *in vitro* optogenetic stimulation of glutamatergic terminals in the NAc, the authors report that sleep deprivation reduces neurotransmission from mPFC but not vHipp, MD, or BLA. The authors also show that sleep deprivation increases sucrose self-administration. Therefore, Liu et al. (2016) implicate the mPFC-NAc pathway in the relationship between loss of sleep and enhanced reward seeking. Lastly, in the same sense of the aforementioned reports (O’Donnell and Grace, 1995; Finch, 1996; Calhoon and O’Donnell, 2013; Liu et al., 2016), striatal inputs have recently gained a comprehensive anatomical map (Hunnicutt et al., 2016). Based on such map, future studies on the convergence of thalamic, cortical, and hippocampal projections to striatal subdivisions may further elucidate sensorimotor and associative functions.

**Figure 2:**
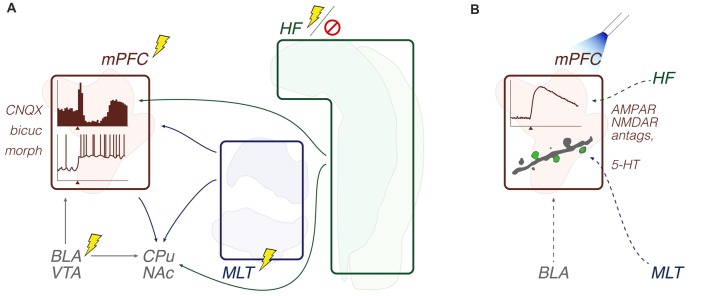
Input convergence: methods and objects of study merged into a schematic summary. The figure portrays generic representations of techniques reviewed in the “Input convergence” subsection. Arrows from the HF are generically positioned, i.e., no distinction is made between dorsal, intermediate and ventral domains. Outlines of brain areas are based on Figure 1. **(A)** *In vivo* electrophysiology, intracerebral electrical stimulation and pharmacological manipulations. Experiments evaluated the prefrontal and striatal concentration of inputs through recording evoked responses upon afferent electrical stimuli, including during drug effects. Lightning and forbidden sign icons represent electrical pulses and hippocampal lesion, respectively. Representative prefrontal cortical data: perievent rate histogram (top) and single-cell voltage trace (bottom) with x-axis arrowheads representing electrical stimuli (e.g., Gigg et al., 1994; Lewis and O’Donnell, 2000). **(B)** *In vitro* electrophysiology, optogenetics and pharmacological manipulations. Experiments evaluated the prefrontal concentration of inputs through recording evoked responses upon optogenetic triggering of glutamate release, including during drug effects. The beam icon represents blue light pulses for stimulation of channelrhodopsin-expressing presynaptic terminals (dashed arrows). Representative prefrontal cortical data: perievent current trace, and two-photon microscopy of a dendrite (gray) with evoked Ca^2+^ signals (green; Little and Carter, 2012). Abbreviations: antags, antagonists; bicuc, bicuculline; BLA, basolateral amygdala; CPu, caudate-putamen; HF, hippocampal formation; MLT, midline/paramidline thalamus; morph, morphine; mPFC, medial prefrontal cortex; VTA, ventral tegmental area.

What emerges from this literature is that limbic thalamic and hippocampal afferents to the mPFC interact with each other, are modulated by interneuronal processing, and are influenced by monoaminergic inputs in complex firing patterns. This combination of events ultimately regulates mPFC outputs to the limbic striatum, which itself integrates several other limbic afferents. Methodologically, the most prevalent strategy consisted of anesthetized single-unit recordings during electrical intracerebral stimulation, eventually along with lesions and drug applications. More recently, optogenetic experiments *in vitro* have also been used to study input drive involving the mPFC (Figure 2; Table 1).

### Synaptic Plasticity: Relevance to Learning and Brain Disorders

In contrast to the preferentially neurophysiological studies of the previous subsection, the synaptic plasticity reports we delimited are biased to behavioral and neuropsychiatric implications. Hugues and Garcia (2007) were the first to directly compare vHipp- and MD-induced synaptic plasticity in the mPFC throughout a learning paradigm: auditory fear conditioning (freezing behavior upon tone-shock pairings) and its extinction (tone-alone trials) over 7 days. Rats were chronically implanted with a recording electrode in the mPFC, and stimulating electrodes in the ipsilateral vHipp, MD, and eyelid for unconditioned stimuli (eyelid shocks). Through evoking fPSPs between tone-alone trials (i.e., during fear extinction), Hugues and Garcia (2007) found opposite dynamics between vHipp- and MD-driven responses: respectively a decrease and an increase in fPSP amplitudes when comparing extinction days 1 and 7. Induction of long-term depression (LTD) through a 2-Hz train into vHipp was able to disrupt this dynamics, in addition to impairing fear extinction. Thus, mPFC inputs can undergo varying learning-associated plasticity processes, whose exogenous disturbances (e.g., train stimulation protocols) are reflected by behavioral alterations.

Chronic electrophysiology studies from another research group (Eleore et al., 2011; López-Ramos et al., 2015) have also evaluated this plasticity-behavior interplay. Eleore et al. (2011) monitored eyeblinks while delivering electric shocks into the trigeminal nerve as unconditioned stimuli. The same chronic implants included a stimulating electrode in the thalamic Re nucleus, and a recording electrode in either mPFC or dorsal CA1. Differently from Hugues and Garcia (2007) and Eleore et al. (2011) observed no changes in paired pulse-evoked fPSPs throughout tone-shock pairings. High-frequency stimulation (HFS) of the Re was also ineffective in inducing long-term potentiation (LTP) in either recorded sites, but did impair the eyeblink conditioning. Also in trigeminal nerve-implanted rats, López-Ramos et al. (2015) used eyelid shocks as negative reinforcements in a food vs. shock decision-making task. For paired-pulse fPSP evoking, a recording electrode was implanted in the mPFC, and stimulation electrodes were placed in either MD, BLA, or temporal CA1. Basically, López-Ramos et al. (2015) observed that fPSP amplitudes reduced with shock intensity and, therefore, behavioral inhibition, regardless of the stimulation site. A conclusion from these studies is that multi-site synaptic plasticity assessments tend to provide apparently conflicting results depending on the behavioral paradigm and stimulated areas, e.g., MD-evoked fPSPs are associated with the extinction of freezing behavior (Hugues and Garcia, 2007), whereas Re-evoked fPSPs seem indifferent to the eyeblink conditioning (Eleore et al., 2011).

Also in chronically implanted rats, Grupe et al. (2014) used auditory stimuli, not intracerebral pulses, to evoke LFP responses. Event-related potentials (ERPs) were chronically recorded from four brain sites, mPFC, MD, temporal CA1 and auditory cortex (Aud Ctx), while rats performed a two-tone (and two-lever) discrimination task. Although ERP signatures expectedly varied across channels, all of them manifested P300-like deflections—positive peaks at ~300 ms latency from stimulus offset—whose amplitudes varied with trial types (target or non-target tone) and operant responses (correct or incorrect). Noteworthy, peroral administration of the “smart drug” modafinil (weak dopamine reuptake inhibitor), or NS9283 (positive allosteric modulator of α4β2 nicotinic receptors) respectively decreased the latency and increased the amplitude of P300 from the MD thalamus and Aud Ctx. However, neither drugs improved the behavioral performance. This apparent dissociation between ERP and behavioral performance under cognitive enhancing compounds should, according to Grupe et al. (2014), encourage new drug discovery designs involving the highly translational P300 measure.

Four other studies have provided support for the MD role in hippocampal-prefrontal fPSP and neuropsychiatric dysfunctions (Kiss et al., 2011a,b; Sloan et al., 2011a,b). Differently from the works above, they were performed under urethane anesthesia. Kiss et al. (2011a) found that inactivation of the rat MD with lidocaine reduces CA1/Sub-mPFC paired-pulse facilitation (PPF), a short-term form of synaptic plasticity (Zucker and Regehr, 2002; Citri and Malenka, 2008). This result could be reproduced by intravenous administration of the psychotomimetic agent MK-801 (NMDA receptor antagonist), implicating the thalamic-prefrontal loop in this drug’s systemic effects. Moreover, LFP recordings from the mPFC revealed that both intra-MD lidocaine and systemic MK-801 disrupts urethane-driven 2 Hz oscillations into a less regular delta rhythm (Kiss et al., 2011a). In a similar work from the same group (Kiss et al., 2011b), abnormal PPF and delta power in the mPFC were replicated when MK-801 was infused into the MD, but not the mPFC itself. Furthermore, intravenous MK-801 induced a net suppression of mPFC unit activity, although a minor proportion of units showed increased firing rate (however, see chronic recordings of Wood et al., 2012). This suggests that acute psychotomimetic effects involve downstream influences to the mPFC, including alterations in the excitatory-inhibitory balance of mPFC neural activity (Kiss et al., 2011b). Sloan et al. (2011a,b) also evaluated the effects of pharmacological inactivation of the MD (either muscimol of tetrodotoxin, TTX) on the hippocampal-prefrontal communication, though using an epilepsy-relevant brain stimulation. Altogether, the two studies (Sloan et al., 2011a,b) demonstrated that MD inactivation reduces the amplitudes of Sub-evoked fPSP in the MD, in addition to suppressing mPFC afterdischarges induced by subicular kindling (20-Hz trains). Therefore, CA1/Sub-mPFC-MD interactions respond to both psychosis-relevant (MK-801) and epilepsy-relevant (subicular kindling) manipulations, suggesting that such circuit participates in both of these dysfunctions.

A more recent work also included fPSP recordings under anesthesia (Jett et al., 2017). Rats previously submitted to chronic unpredictable stress and their controls were anesthetized with chloral hydrate for implantation of recording (mPFC) and stimulating electrodes (MD or vHipp). Through analyzing current-response curves (i.e., input-output (I/O) curves), Jett et al. (2017) found that the MD-mPFC recruitment is selectively weakened in stress-exposed rats. The study also reports that chronic stress impairs the prefrontal expression of c-Fos induced by MD pharmacological activation, among other findings.

**Figure 3:**
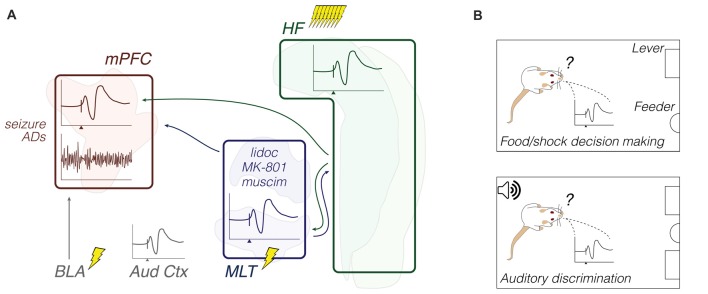
Synaptic plasticity: methods and objects of study merged into a schematic summary. The figure portrays generic representations of techniques reviewed in the “Synaptic plasticity” subsection. Arrows from the HF are generically positioned, i.e., no distinction is made between dorsal, intermediate and ventral domains. Outlines of brain areas are based on Figure 1. **(A)** *In vivo* electrophysiology, intracerebral electrical stimulation, and pharmacological manipulations. Experiments evaluated the prefrontal and auditory cortical field potential reactivity to afferent electrical stimuli or sound stimuli, including during drug effects and after induction of synaptic plasticity or seizure activity. Lightning icons represent single electrical pulses, or trains of pulses. Representative prefrontal cortical voltage traces: evoked (top) and spontaneous field potentials (bottom), with the *x*-axis arrowhead representing afferent stimuli (e.g., Hugues and Garcia, 2007; Kiss et al., 2011b). Field potential responses recorded from other brain sites are also illustrated (e.g., Grupe et al., 2014). **(B)** Chronic electrophysiology (field potential responses) during a food vs. trigeminal shock operant task (top), and a two-lever auditory discrimination task (bottom). Experiments evaluated relationships between behavioral performance and evoked field potential responses (e.g., López-Ramos et al., 2015). Abbreviations: ADs, after-discharges; Aud Ctx, auditory cortex; BLA, basolateral amygdala; HF, hippocampal formation; lidoc, lidocaine; MLT, midline/paramidline thalamus; mPFC, medial prefrontal cortex; muscim, muscimol.

In comparison with the previous subsection, these field electrophysiology studies are more heterogeneous in their findings and speculations. The results from different stimulation sites and conditioning procedures suggest, at most, that the strength and direction of synaptic plasticity (e.g., LTP, LTD, or no change) in specific axonal pathways (e.g., MD-mPFC or CA1/Sub-mPFC) may dynamically vary throughout learning. ERP across the mPFC and related regions are sensitive to both sensory cues and cognitive-enhancing drugs. Psychotomimetic drug effects on the hippocampal-prefrontal communication, and the ability of this pathway to spread seizures may both depend on the limbic thalamus. Finally, MD-mPFC (but not vHipp-mPFC) I/O curves seem affected by pre-exposure to stress. Methodologically, chronic electrophysiology studies included Pavlovian fear conditioning/extinction and operant lever press tasks, while psychosis- and epilepsy-relevant studies were undertaken during urethane anesthesia (Figure 3; Table 1).

### Functional Coupling

#### Transthalamic Synchrony: From Functional Development to Adult Navigation

Both the anatomical and electrophysiological literature point to the dorsal and ventral midline thalamic areas as key regulators of the mPFC activity. A sequence of recent reports indicates that the ventral midline nuclei, in particular, tend to draw attention due to their direct roles in the prefrontal-hippocampal, and hippocampal-prefrontal coupling (Ito et al., 2015; Hallock et al., 2016; Hartung et al., 2016; Zimmerman and Grace, 2016; Roy et al., 2017; Kafetzopoulos et al., 2018). We begin with Hartung et al. (2016), who examined these interactions from the functional development perspective. In two cohorts of neonatal rats (P7–9) under urethane anesthesia, the authors recorded LFP and unit activity from either mPFC (layers 2/3), temporal CA1 (stratum pyramidale), and Re/Rh nuclei, or mPFC, temporal CA1 and lateral EC (layers 2/3). Using polar histograms, theta amplitude envelopes (4–12 Hz), and firing rate histograms, Hartung et al. (2016) initially described tight relationships between theta bursts and unit activity in both Re/Rh and EC. Then, the authors employed coherence and cross-correlation analyses of theta-filtered data to compare Re/Rh and EC roles in modulating the mPFC and CA1. Basically, results indicate that the thalamic control over CA1 is monosynaptically driven by the mPFC, while the entorhinal control over CA1 is not. As reviewed below, this transthalamic feedback between mPFC and CA1 is important for the adult spatial working memory. Because a similar feedback system seems already present in P7–9 pups, it could serve as a template for the adult cognitive performance, as proposed by Hartung et al. (2016).

Further to Hartung et al. (2016), LFP synchronization across the prefrontal-thalamic-hippocampal route has been shown to be influenced by ascending projections from the brainstem. Roy et al. (2017) used urethane-anesthetized rats for recording LFP from mPFC, Re, and the dorsal hippocampus (dHipp, according to the authors’ stereotaxic coordinates). In the same preparation, the pontine reticular formation (RF) was electrically stimulated with 100 Hz trains at different pulse intensities. As reported by Roy et al. (2017), 2 Hz-peaked mPFC delta and 8 Hz-peaked hippocampal theta were respectively reduced and increased with the intensity of pontine stimulation. Recordings from Re showed a combination of both effects, and inactivation of the Re with lidocaine was able to decrease delta, but not theta, coherence between mPFC and hippocampus. These findings are in apparent contradiction with those of Hartung et al. (2016): while Roy et al. (2017) found that mPFC-CA1 theta coherence is minimally sensitive to Re inactivation, Hartung et al. (2016) found that mPFC and CA1 are functionally coupled via Re theta bursts. Variables such as postnatal age, electrode positioning, silencing of passing fibers by lidocaine, and pontine stimulation possibly account for the inconsistency between studies. It is nevertheless clear that the anatomical relationships of the ventral midline thalamus with both the mPFC and hippocampus (Prasad and Chudasama, 2013; Varela et al., 2014) are reflected by LFP measures of connectivity.

Behavioral outcomes of this functional connectivity are just emerging. This is the case of Ito et al. (2015), who recorded trajectory-dependent firing during performance on modified T-mazes. Using rats trained in a continuous alternation task, Ito et al. (2015) compared firing patterns from two dHipp subfields: CA1 (which receives afferents from Re) and CA3 (which does not). Both CA1 and CA3 place cells increased their firing rates with the subjects approaching the decision point of the T-maze stem, i.e., the junction between side arms. However, only CA1 cells responded with different rates depending on the arm to be chosen. Interestingly, decision-related activity patterns were also observed in Re and mPFC, and Re inactivation with either ibotenic acid lesion or optogenetic silencing attenuated trajectory-dependent activity in CA1. Thus, among several other electrophysiological and behavioral analyses, Ito et al. (2015) revealed forward-oriented neural representations in the mPFC-Re-CA1 system, in contrast to the mere place-cell activity observed in CA3. Synchrony in the opposite direction, CA1-thalamus-mPFC, has also been reported to underlie T-maze performance. Hallock et al. (2016) trained tetrode-implanted rats (dorsal CA1 and mPFC) to switch between two spatial tasks: working memory-independent delayed alternation (without sensory cues: bare T-maze floor) and working memory-dependent conditional discrimination (with sensory cues: either mesh or wood floor inserts). Briefly, Hallock et al. (2016) described working memory-specific patterns of: mPFC neuronal ensemble encoding, mPFC single-unit entrainment to hippocampal theta activity, and CA1-mPFC theta phase coherence, always in relation to T-maze locations (start point, stem and choice point). In another experiment, the authors found that inactivation of the Re/Rh with muscimol disrupts these measures of CA1-mPFC synchrony. Consistently with both Ito et al. (2015) and Hallock et al. (2016), Kafetzopoulos et al. (2018) report that Re lesion impairs CA1-mPFC coherence in the theta and beta bands. In separate non-electrophysiological experiments, the same authors found that Re lesion produces antidepressant-like effects (forced swim and sucrose preference tests) and prevents mPFC dendritic alterations caused by chronic mild stress. Thus, contrasting behavioral paradigms such as T-maze, forced swim, and sucrose preference share the Re/Rh area as an anatomical correlate. This suggests that the ventral midline thalamic role in both the hippocampal-prefrontal communication and cognitive/emotional processes is still underexplored.

A more complicated scenario may arise when considering the interactions between transthalamic circuits and subcortical neuromodulatory centers, such as the VTA. Using chloral hydrate-anesthetized rats, Zimmerman and Grace (2016) described gating actions of the Re nucleus on ventral Sub- and mPFC-related dopamine neuron activity. More specifically, pharmacological activation of the Re with NMDA increased the population activity among VTA dopamine neurons, which was prevented by TTX injection into the ventral Sub. A higher population activity in the VTA was also achieved through TTX inactivation of the mPFC, which was reverted by TTX inactivation of the Re. Finally, in a separate group of awake Re-cannulated rats, Zimmerman and Grace (2016) showed that intra-Re NMDA potentiates a psychotic-like behavior: amphetamine-elicited hyperlocomotion on the open field (OF). As discussed by the authors, subicular and prefrontal outputs may balance the Re-VTA drive, whose perturbation could contribute to the dopaminergic and thalamocortical dysfunctions under schizophrenia symptoms. Noteworthy, these VTA recordings of Zimmerman and Grace (2016) were performed more than a decade after the VTA stimulation studies cited previously (Lewis and O’Donnell, 2000; O’Donnell et al., 2002; Floresco and Grace, 2003), indicating the necessity of more VTA studies within the hippocampal-prefrontal-thalamic framework.

**Figure 4:**
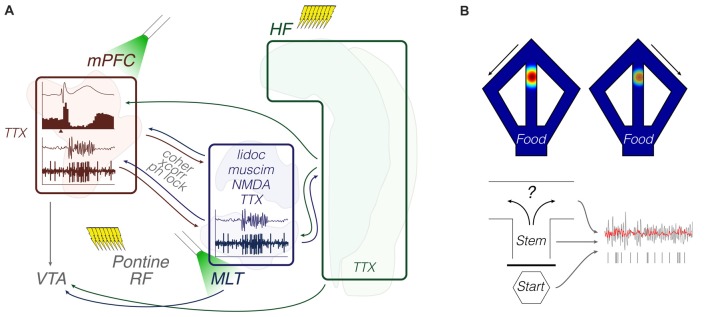
Functional coupling: methods and objects of study merged into a schematic summary. The figure portrays generic representations of techniques reviewed in the “Functional coupling” subsection. Arrows from the HF are generically positioned, i.e., no distinction is made between dorsal, intermediate and ventral domains. Outlines of brain areas are based on Figure 1. **(A)** *In vivo* electrophysiology, intracerebral electrical/optical perturbation and pharmacological manipulations. Experiments evaluated response evoking and circuit connectivity through intra-cerebral drugs and afferent stimulation, including optogenetic terminal inhibition. Lightning icons represent trains of pulses. The beam icon represents intracerebral green light pulses for activation of archaerhodopsins, including on presynaptic terminals. Representative prefrontal cortical data: field potential response with corresponding perievent firing histogram (top; *x*-axis arrowhead representing afferent stimuli), and theta-filtered voltage trace with corresponding multi-unit activity (bottom; e.g., Hartung et al., 2016; Padilla-Coreano et al., 2016; Bueno-Junior et al., 2018). The latter is also illustrated in the MLT. **(B)** Chronic electrophysiology and representative activity patterns during maze performance. Top: place-cell and trajectory-dependent firing. Bottom: illustrative single-unit entrainment to raw (gray) and filtered (red) field oscillations depending on the maze location or trial phase (e.g., Ito et al., 2015; Hallock et al., 2016; Bolkan et al., 2017). Abbreviations: coher, coherence; HF, hippocampal formation; lidoc, lidocaine; mPFC, medial prefrontal cortex; MLT, midline/paramidline thalamus; muscim, muscimol; ph lock, phase locking; RF, reticular formation; TTX, tetrodotoxin; VTA, ventral tegmental area; xcorr, crosscorrelation.

In summary, the ventral midline thalamus seems as important as the EC in mediating flows of information between the mPFC and HF, already from early life. Activity synchronization in cognition-relevant bands, such as theta, is related to brainstem activity and spatial working memory, which in turn is represented by trajectory-dependent patterns across the prefrontal-thalamic-hippocampal system. Derangements in specific subcircuits of this system may culminate in unbalanced thalamic drive onto VTA dopamine cells, possibly contributing to dysfunctional behaviors. Although far from conclusive, this emerging literature points to a ventral midline thalamus research trend. Methodologically, anesthetized recordings were used to explore either firing responses to pharmacological stimulation, or fine LFP patterns, including during pontine-driven theta activity. Chronic recordings were made during T-maze performance. Together with pharmacological or optogenetic manipulations of the Re/Rh, T-maze experiments yielded a wealth of data on decision making, ensemble encoding, and oscillatory coherence (Figure 4; Table 1).

#### Network Plasticity, Input Dissociation and Thalamocortical Amplification: Relevance to Cognition and Anxiety

More studies are needed to jointly investigate the issues of the previous subsections, namely input convergence, synaptic plasticity, and transthalamic communication. Exploring these relationships may further elucidate how afferent-driven firing (Gigg et al., 1994; Floresco and Grace, 2003) interacts with the plasticity of LFP responses (Hugues and Garcia, 2007) throughout homeostatic and mnemonic processes (Tononi and Cirelli, 2003; Levenstein et al., 2017).

An investigation from our group is an attempt in this direction. Using chronically implanted rats, Bueno-Junior et al. (2018) delivered electrical paired pulses (every 10 s) into the temporal CA1/Sub, while recording single-unit activity and fPSP from mPFC and PV/MD. Paired pulse-locked excitatory responses (<50 ms latency) were found in a proportion of mPFC and PV/MD units, followed by transient suppressions of their activities (160–400 ms latency). Particularly in the mPFC, secondary excitatory responses were observed (>400 ms latency), and they were potentiated after LTP induction, i.e., HFS of CA1/Sub. In complementary anesthetized recordings (Bueno-Junior et al., 2018), archaerhodopsin-transfected rats received the same implants, except for an optrode in PV/MD. CA1/Sub paired pulses were delivered as in the main experiment, but this time they were randomly accompanied by PV/MD light pulses (50% probability), in order to probe the thalamic role in the CA1/Sub-mPFC recruitment. We found that the LTP-related >400 ms excitation of the mPFC is attenuated by PV/MD light pulses. Therefore, we speculate that CA1/Sub-mPFC inputs unfold into an excitatory resonance that is plastic and partially dependent on the mPFC-PV/MD loop.

Prior to Bueno-Junior et al. (2018), a series of three studies from one research group reported MD-mPFC input perturbation using chemogenetic (Parnaudeau et al., 2013) or optogenetic tools in behaving mice (Padilla-Coreano et al., 2016; Bolkan et al., 2017). Parnaudeau et al. (2013) used the designer drug clozapine-N-oxide (CNO) to reduce MD firing rate during a behavioral flexibility lever-press task (reversal learning) and a working memory T-maze task (delayed nonmatch-to-sample, DNMS). In both tasks the authors observed impaired performance upon MD inhibition. Then, through recording LFP across DNMS trials, Parnaudeau et al. (2013) found that task acquisition was commensurate with an increase in beta-range MD-mPFC coherence, and this effect was weaker in CNO-treated mice. The authors additionally report that MD inhibition selectively disrupts beta-range phase locking between MD firing and mPFC oscillations during the DNMS task, while sparing the same measure between MD and dHipp. Thus, chemogenetic silencing effects during DNMS were specific to the MD-mPFC connectivity. A comparable dissociation between MD and hippocampus is reported by Padilla-Coreano et al. (2016). Through delivering light pulses in the mPFC of mice, they optogenetically inhibited archaerhodopsin-transfected terminals from either MD or vHipp during three anxiety tests: EPM, OF, or novelty suppression feeding. Padilla-Coreano et al. (2016) found anxiolytic effects upon vHipp-mPFC, but not MD-mPFC, terminal inhibition. Then, the authors observed that vHipp-mPFC terminal inhibition disrupts: (1) phase locking of mPFC single units to vHipp, but not BLA, theta oscillations; and (2) mPFC firing patterns underlying the preference for open or closed arms of the EPM, in addition to other results.

The hippocampal-prefrontal and thalamic-prefrontal cooperation was more directly assessed in the third work of this series (Bolkan et al., 2017). First, the authors revealed that each direction of the MD-mPFC loop (top-down or bottom-up) subserves a particular cognitive function. This dissociation was made possible using the T-maze DNMS task, and optogenetic disruption of presynaptic activity during specific task phases. Impaired performance was observed with either MD-mPFC terminal inhibition during delay phases, or mPFC-MD terminal inhibition during choice phases. Consistently, the functional directionality between thalamic beta oscillations and prefrontal unit activity was observed to shift across trial phases. Then, among other results, Bolkan et al. (2017) report that optogenetic inhibition of distinct afferents—either from MD or vHipp—differentially affects mPFC unit activity depending on the trial phase. More specifically, MD-mPFC or vHipp-mPFC terminal inhibition disrupted delay-related or spatially-related activity patterns, respectively. Thus, each mPFC input preferentially contributes to a certain aspect of working memory: from delay-related activity maintenance to spatial encoding. Altogether, these three studies (Parnaudeau et al., 2013; Padilla-Coreano et al., 2016; Bolkan et al., 2017) comprise a wealth of strategies, ranging from activity measures (e.g., MD-dHipp or vHipp-mPFC phase locking) to circuit manipulations (e.g., inhibition of MD cells or their efferent terminals) and behavioral paradigms (e.g., DNMS cognition or EPM anxiety). This multiplicity of strategies correspondingly reflects a multiplicity of implications, which we outline in the next section.

In parallel to Bolkan et al. (2017), another research group (Schmitt et al., 2017) was also able to decompose specific aspects of a cognitive task (two-alternative forced choice) and their neural representations. First, the authors report different mPFC firing patterns under different attention-guiding rules (i.e., attend to vision or attend to audition). Then, the authors show that optogenetic excitation of the MD during delay periods of the task enhances both the behavioral performance and the underlying mPFC firing patterns. As discussed by Schmitt et al. (2017) and subsequent review articles from the same group (Halassa and Kastner, 2017; Nakajima and Halassa, 2017; Rikhye et al., 2018), such MD-mPFC relationships represent a cognition-relevant amplification system. Through this kind of system, ascending thalamic projections seem able to both shift and sustain intracortical computations depending on the cognitive demand, rather than just relaying information to or between cortical areas. Interestingly, these studies do not emphasize hippocampal outputs, and hence they were not included in Table 1. This reinforces that the long-studied MD-mPFC loop is still attractive for research, especially if considering its role in the hippocampal-prefrontal communication.

Lastly, we mention another chronic electrophysiology study (Hernández-González et al., 2017), in which multiple sites were recorded while rats performed a touch-screen operant conditioning task. First the authors identified a specific LFP pattern in the mPFC (transient decrease in theta and increase in gamma power) predicting a goal-directed behavior (going to the screen to nose-poke it). Then, among other analyses, they evaluated LFP coherence between mPFC and five interconnected areas (primary motor cortex, MD thalamus, VTA, NAc, and CA1). They found that this behavior-predicting mPFC pattern is preferentially coherent with the primary motor cortex, MD, and VTA (in this order of importance), suggesting that functional connectivity between mPFC and this subset of afferents anticipates the touch-screen response. In addition, using a brain-machine interface, Hernández-González et al. (2017) found that this same mPFC pattern can trigger a visual cue on the touch screen upon training. Therefore, power and coherence predictors of goal-directed behaviors can be useful in brain-machine interfaces, which points to interesting research possibilities involving executive circuits.

In summary, the reports described in this subsection suggest that hippocampal-prefrontal pathways and thalamic-prefrontal loops interact with each other in plasticity- and cognition-relevant manners. These reports also indicate that combining multi-site recordings with pathway-specific manipulations can be highly informative, either using purely neurophysiological approaches, or mechanistically dissecting working memory, behavioral flexibility, anxiety, and goal-directed behaviors. Methodologically, these studies employed *in vivo* recordings during electrical paired-pulse recruitment, electrical induction of LTP, chemogenetic or optogenetic control (including presynaptic terminal inhibition), DNMS performance, anxiety tests, and operant conditioning (Figure 4; Table 1).

## Critical Synthesis and Research Directions

**Figure 5:**
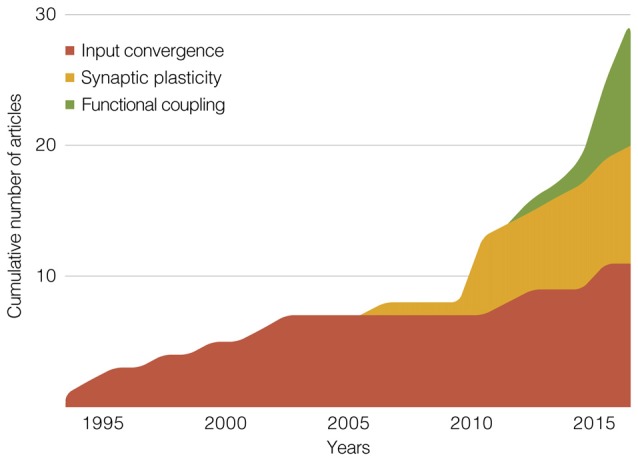
Stacked area graph showing the cumulative number of publications within our scope. The graph depicts three methodological categories of studies that jointly manipulated/recorded hippocampal, prefrontal and thalamic sites. Summaries of these categories make up the main section of this review article: “Rodent Electrophysiology Studies.” The *x*-axis is divided in 1-year bins, from 1994 to 2017.

Our attempt with the previous section was to merge a historical line with a methodological categorization. Two findings from this effort are illustrated in Figure 5: an exponential-like growth in the cumulative number of articles, and a chronological pattern across the proposed categories. As shown by Figure 5 (and also Table 1), input convergence studies predominated until 2011, from when the proportion of synaptic plasticity and functional coupling studies has rapidly expanded. We recognize that methodological boundaries can be uncertain. This is especially true among works from the last 5 years, which included multi-channel recordings during various behavioral paradigms, multiple preparations within the same study (e.g., chronic, *in vitro*, or anesthetized recordings), and modern tools of brain stimulation (e.g., optogenetics). Nevertheless, it is interesting to note that we could clarify methodological trends, which will possibly assist researchers who are looking for insights into their next experiment.

We also recognize that non-methodological themes, e.g., cognition mechanisms or neuropsychiatric disorders, could have been used to organize the review. However, this kind of approach is already covered by other reviews, and the reader is referred to them (e.g., Lisman, 2012; Godsil et al., 2013; Cassel and Pereira de Vasconcelos, 2015; Griffin, 2015; Pergola et al., 2015; Wolff et al., 2015; Sigurdsson and Duvarci, 2016; Parnaudeau et al., 2018). Still, we outline below some general implications and interrogations.

### Learning, Anxiety and Navigation

**Figure 6:**
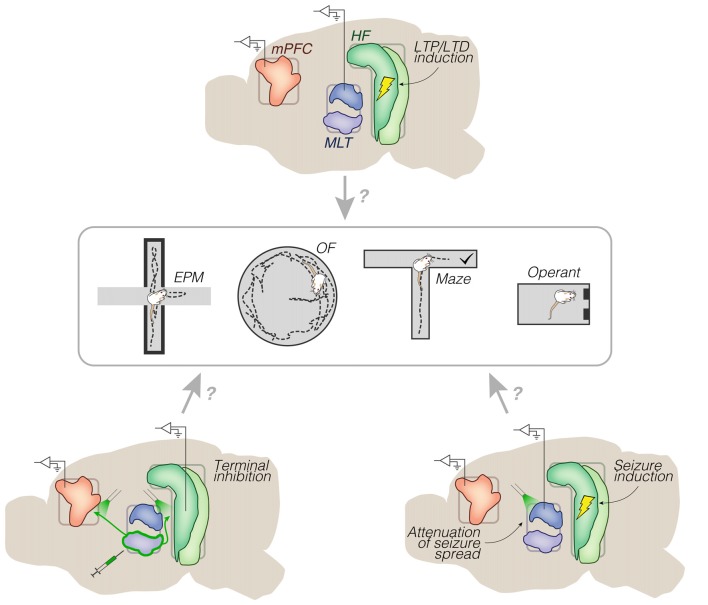
Representative research directions. Chronic implants (mid sagittal views) surrounding a set of behavioral testing options. Top: electrical trains of pulses into the HF, and monitoring of long-term effects on spontaneous and/or evoked activity (e.g., from prelimbic cortex or dorsal midline thalamus). No distinction is made between dorsal, intermediate and ventral domains of the HF. Bottom left: optogenetic inhibition of archaerhodopsin-transfected efferents from the ventral midline thalamus. Bottom right: optogenetic attenuation of electrically-induced seizures from the HF. These implant strategies could be used in animal models of brain disorders, including schizophrenia and epilepsy. Abbreviations: EPM, elevated plus maze; HF, hippocampal formation; LTD, long-term depression; LTP, long-term potentiation; MLT, midline/paramidline thalamus; mPFC, medial prefrontal cortex; OF, open field.

Electrically-evoked fPSP, which are standard in synaptic plasticity studies since Bliss and Lømo (1973), allow measuring pathway recruitment and train stimulation effects, in addition to serving as neurophysiological markers of learning. As implied by a set of reviewed studies (Hugues and Garcia, 2007; Eleore et al., 2011; López-Ramos et al., 2015), there is room for exploring both stimulation- and learning-induced plasticity of prefrontal fPSP. Altogether, these studies show that: synaptic efficacy in CA1-mPFC and MD-mPFC projections follow opposite dynamics throughout fear extinction in an LTD-dependent manner; both Re-mPFC and Re-CA1 projections participate in aversive conditioning in an LTP-independent manner; and both CA1-mPFC and MD-mPFC projections are sensitive to reward vs. aversion conflicts. As it can be seen, it is difficult to draw firm conclusions from these data. Hence, although these are methodologically comparable reports, clearly there is a need for further homogenization across future studies, like fixing the behavioral or stimulation variable (e.g., fear extinction, LTD, etc.) while exploring different neuroanatomical substrates (e.g., dorsal vs. ventral mPFC, MD vs. Re, medial vs. lateral MD, etc.), or vice-versa (Figure 6).

Conversely, converging results can be noticed from methodologically distant experiments. Using chronic electrophysiology, Padilla-Coreano et al. (2016) found that optogenetic inhibition of vHipp-mPFC (but not MD-mPFC) terminals reduces EPM anxiety and theta phase locking. In turn, Kjaerby et al. (2016) used *in vitro* electrophysiology to demonstrate that optogenetic excitation of vHipp-mPFC (but not MD-mPFC) terminals is suppressed via serotonergic 5-HT1B receptors; then in a chronic electrophysiology experiment, Kjaerby et al. (2016) found that 5-HT1B agonism reduces EPM anxiety and mPFC theta power, which is generally consistent with Padilla-Coreano et al. (2016). In fact, two studies without thalamic manipulation/recording (Adhikari et al., 2010, 2011) had previously shown that vHipp-mPFC theta activity distinguishes between fear and safety, which was demonstrated to additionally involve the BLA (Likhtik et al., 2014; Stujenske et al., 2014). In contrast to the heterogeneous scenario from fPSP and learning reports, these studies agree that the level of vHipp-mPFC (but not MD-mPFC) theta activity predicts the level of anxiety. Thus, anxiety-related theta may be used as a hallmark in future LTP/LTD experiments on: innate anxiety, the ability to extinguish learned fear, the ability to distinguish safety from danger, the thalamic vs. amygdalar involvement in each of these behaviors, and the efficacy of stimulation protocols for separately modulating them (Figure 6).

Stimulation protocols for dissecting behavioral components can include synaptic plasticity paradigms (e.g., HFS and theta bursts), which consist of patterned sequences of trains for modulating a two-node axonal pathway. However, behavioral dissections can also probe three- or four-node circuits using stimuli that are sub-threshold for inducing LTP/LTD (e.g., a single pulse/train every 10–30 s). Bueno-Junior et al. (2018) combined these two approaches, though without collecting behavioral data. Electrically-evoked firing responses in the CA1/sub-mPFC pathway (elicited every 10 s) were initially shown to be potentiated by HFS in freely-moving rats. Then in complementary anesthetized recordings without HFS, CA1/sub stimuli (also every 10 s) were either accompanied or not by thalamic optogenetic perturbation. The main finding was that CA1/sub stimuli with thalamic light on elicited weaker mPFC responses. While these results are insightful for the neurophysiology of CA1/sub-mPFC-PV/MD interactions, they are limited by the lack of behavioral correlates. An opposite situation is represented by other reviewed studies (Ito et al., 2015; Bolkan et al., 2017), which involved high-precision multi-site probing during behavioral testing without induction of LTP/LTD. Ito et al. (2015) found neural representations of T-maze trajectories in the mPFC. Then, through optogenetically silencing the Re nucleus during T-maze trials, they were able to disrupt similar trajectory-related activities in the dorsal CA1. In turn, Bolkan et al. (2017) were able to dissociate neural representations of different aspects of a T-maze DNMS task using optogenetic terminal inhibition. In particular, spatial encoding, delay periods, and choice phases were preferentially associated with vHipp-mPFC, MD-mPFC and mPFC-MD communications, respectively.

As judged by this set of articles (Ito et al., 2015; Bolkan et al., 2017; Bueno-Junior et al., 2018) as well as the thalamic pharmacological inhibition study of Hallock et al. (2016), diversifying stimulation designs while focusing on a behavioral paradigm (e.g., T-maze) can lead to multiple experiments. Particularly, the different connectivity patterns of PV/MD and Re/Rh (Hoover and Vertes, 2007; Vertes et al., 2015) imply that each of these nuclei may preferentially subserve distinct behavioral constituents, like spatial encoding, working memory maintenance, decision-making, or specific combinations among them. Thus, implanting into both PV/MD and Re/Rh in the same subjects, then randomly stimulating/inhibiting these areas across trials (e.g., PV/MD alone, Re/Rh alone, or combined) could seize control of a substantial portion of all possible hippocampal-prefrontal-thalamic interactions, at least in the chosen behavioral test. Another possibility would be to transfect the Re/Rh for optogenetic co-stimulation (or co-inhibition) of Re/Rh-mPFC and Re/Rh-CA1 terminals (Figure 6). Based on these strategies, many questions could be raised. Can the thalamic-prefrontal resonance preferentially recruit either PV/MD or Re/Rh contingent upon behavioral demands? Are prospective representations of maze trajectories confined to Re/Rh-related sub-circuits, or are they also modulated by the mPFC-PV/MD loop? Are these processes sensitive to LTP/LTD induction? The same rationale is applicable to the septal-temporal axis of the HF (O’Neill et al., 2013), as well as the dorsal-ventral extent of the mPFC (i.e., from the anterior cingulate to the infra-limbic area): more studies are needed to further dissociate hippocampal and prefrontal subdivisions in terms of cognitive roles. To increase the challenge even further, distinct behaviors can share common electrophysiological markers. For example, different oscillatory patterns in the vHipp-mPFC pathway are associated with different aspects of T-maze cognition: O’Neill et al. (2013) linked theta activity to working memory, whereas Spellman et al. (2015) linked gamma activity to cue encoding. As discussed above, vHipp-mPFC theta activity also underlies anxiety behaviors (Adhikari et al., 2010, 2011; Kjaerby et al., 2016; Padilla-Coreano et al., 2016). Therefore, theta (rather than gamma) oscillations may jointly underlie working memory and anxiety (Jones and Wilson, 2005; Fujisawa and Buzsáki, 2011; Roy et al., 2017; Korotkova et al., 2018). This commonality is consistent with the notion that anxiety and cognitive flexibility are inversely related (Park and Moghaddam, 2017). Therefore, manipulating the anxiety-cognition balance through co-stimulation designs and/or induction of synaptic plasticity may motivate new experiments, including in animal models of innate/learned anxiety, and during drug effects (Figure 6).

### Neuropsychiatric Disorders

Similarly to research on learning, anxiety, and navigation, the study of brain disorders still has much to gain from circuit-level experiments. Some of the reviewed articles are within the framework of schizophrenia (Lewis and O’Donnell, 2000; Kiss et al., 2011a,b; Zimmerman and Grace, 2016), and future research may build upon them. Kiss et al. (2011a,b) report that systemic administration of a psychotomimetic drug (MK-801, NMDA receptor antagonist) disrupts CA1/sub-mPFC electrical recruitment and urethane-driven delta oscillations. Of note, intra-MD (but not intra-mPFC) MK-801 culminated in the same effects, suggesting that hippocampal-prefrontal alterations in psychosis may be partially downstream from thalamic dysfunctions. Consistently, the co-participation of these circuits in human schizophrenia has been reviewed by many authors (e.g., Lisman, 2012; Pergola et al., 2015; Sigurdsson and Duvarci, 2016; Parnaudeau et al., 2018); among the non-behavioral symptoms that predict the transition to schizophrenia is the decreased thalamo-prefrontal functional connectivity, and the increased hippocampal metabolism. Other factors are also implicated by the rodent literature we reviewed, including dysfunctional dopaminergic modulation of the mPFC and its afferents (Lewis and O’Donnell, 2000), and the Re thalamic role in such dysfunction (Zimmerman and Grace, 2016). These studies, along with Kiss et al. (2011a,b), point to several lines of research. In fact, these electrophysiological data are from anesthetized recordings. Thus, opportunities exist for evaluating hippocampal-prefrontal-thalamic subcircuits in rodents with both schizophrenia-like symptoms and chronic implants. Symptom induction methods could range from the perturbation of fetal or adolescent development (e.g., mitotoxin injection during pregnancy, and repeated exposure to cannabinoids) to genetic models (e.g., 22q11.2 microdeletion, and mutations in the DISC_1_ gene). Once adults, subjects could be examined for open-field locomotion, novel object recognition, or maze performance (Figure 6), among other behavioral tests used in conjunction with animal models of schizophrenia (Ruggiero et al., 2017).

The study of seizure propagation could also benefit from circuit-level approaches like those discussed here. Thalamic nuclei can be manipulated for alleviating different forms of pharmacoresistant epilepsy, as suggested by clinical (Osorio et al., 2007) and experimental (Paz et al., 2013) developments in closed-loop deep brain stimulation. Temporal lobe epilepsy, however, is yet to be systematically investigated in this sense. According to two reviewed studies (Sloan et al., 2011a,b) and other publications from the same group (e.g., Bertram, 2014; Zhang and Bertram, 2015), limbic thalamic nuclei are able to amplify seizures from the temporal HF toward the PFC. Thus, closed-loop interfaces involving these thalamic nuclei could be used during the chronic phase of experimental epilepsy, for example after pharmacological or electrical induction of status epilepticus in rodents (Kandratavicius et al., 2014). The efficacy of seizure suppression could then be evaluated through electrophysiological and behavioral analyses. Moreover, the same epileptic subjects could be examined for their interictal behavior, and their propensity to develop comorbid behavioral alterations. For instance, assessing working memory, open-field locomotion, and sensorimotor gating in between seizure monitoring sessions (as in Wolf et al., 2016) could shed light on the long-term consequences of seizures on PFC neural activity (Figure 6). As previously reviewed (Kandratavicius et al., 2012), the cognitive and psychotic comorbidities of temporal lobe epilepsy may derive from hippocampal hyperexcitability, disinhibition of mesolimbic dopamine neurons (Lodge and Grace, 2007; Cifelli and Grace, 2012), and downstream perturbations to extra-temporal circuits, including thalamic-prefrontal loops. Also, seizure incidence has been linked to aberrant innervation from the thalamic reticular nucleus to the midline thalamus (Wolf et al., 2016). This implicates the GABA-driven modes of thalamocortical activity (especially burst firing) in temporal lobe epilepsy, in addition to absence epilepsy (Steriade, 2005). Therefore, seizures are associated with anatomically distributed neurophysiological abnormalities, which deserves further assessment within the hippocampal-prefrontal-thalamic scope.

These research directions are biased to recurrent themes within the delimited literature, which however contains experiments on several other topics. For instance: effects of neonatal hippocampal lesion on biophysical properties of the adult mPFC (O’Donnell et al., 2002), reversal learning during chemogenetic inhibition of the MD (Parnaudeau et al., 2013), effects of cognitive-enhancing drugs on multi-site ERP recordings (Grupe et al., 2014), prefrontal and accumbal role in the sleep-motivation relationship (Liu et al., 2016), maturation of electrophysiological correlates of cognition (Hartung et al., 2016), brain-machine interfaces during operant conditioning (Hernández-González et al., 2017), and effects of chronic stress on mPFC glutamate transmission and dendritic morphology (Jett et al., 2017; Kafetzopoulos et al., 2018), not to mention the anesthetized recordings that initially described mPFC input convergence (e.g., Gigg et al., 1994; Floresco and Grace, 2003). As suggested by the cumulative graph of Figure 5, the next decade may be of great diversification of themes and approaches, including: manipulations of non-rapid-eye-movement sleep rhythms (i.e., cortical slow oscillations, thalamocortical spindles, and hippocampal sharp-wave ripples), as in Latchoumane et al. (2017); further exploration of efference copies traveling via limbic thalamic nuclei (Sherman, 2016; Ouhaz et al., 2018); and the participation of limbic thalamic nuclei in the systems-level memory consolidation (Pereira de Vasconcelos and Cassel, 2015), in a possible expansion of the hippocampal-cortical view of mnemonic organization (Frankland and Bontempi, 2005).

## Concluding Remarks

This historical-methodological narrative gathers rodent electrophysiology studies that directly assessed the hippocampal-prefrontal-thalamic cooperation. During the literature search, common approaches were gradually identified, resulting in the proposal of methodological categories: from purely electrophysiological works of the 1990s to multi-disciplinary investigations in the few years preceding this review article. By offering this summary along with some syntheses and directions, we expect to contribute to experiment designing in the near future.

## Author Contributions

LB-J wrote the manuscript, made the table and figures. JL revised the manuscript.

## Conflict of Interest Statement

The authors declare that the research was conducted in the absence of any commercial or financial relationships that could be construed as a potential conflict of interest.
